# Competitive Perceptrons: The Relevance of Modeling New Bioinspired Properties Such as Intrinsic Plasticity, Metaplasticity, and Lateral Inhibition of Rate-Coding Artificial Neurons

**DOI:** 10.3390/biomimetics8080564

**Published:** 2023-11-23

**Authors:** Diego Andina

**Affiliations:** Grupo de Automatización en Señal y Comunicaciones, Escuela Técnica Superior de Ingenieros de Telecomunicación, Universidad Politécnica de Madrid, 28040 Madrid, Spain; d.andina@upm.es

**Keywords:** neuroplasticity, artificial metaplasticity, intrinsic plasticity, lateral inhibition, winner takes-it-all learning rule, laterally inhibited perceptron, competitive perceptron

## Abstract

This article supports the relevance of modeling new bioinspired properties in rate-coding artificial neurons, focusing on fundamental neural properties rarely implemented thus far in artificial neurons, such as intrinsic plasticity, the metaplasticity of synaptic strength, and the lateral inhibition of neighborhood neurons. All these properties are bioinspired through empirical models developed by neurologists, and this in turn contributes to taking perceptrons to a higher potential level. Metaplasticity and intrinsic plasticity are different levels of plasticity and are believed by neurologists to have fundamental roles in memory and learning and therefore in the performance of neurons. Assuming that information about stimuli is contained in the firing rate of the connections among biological neurons, several models of artificial implementation have been tested. Analyzing their results and comparing them with learning and performance of state-of-the-art models, relevant advances are made in the context of the developing Industrial Revolution 4.0 based on advances in Machine Learning, and they may even initiate a new generation of artificial neural networks. As an example, a single-layer perceptron that includes the proposed advances is successfully trained to perform the XOR function, called the Competitive Perceptron, which is a new bioinspired artificial neuronal model with the potential of non-linear separability, continuous learning, and scalability, which is suitable to build efficient Deep Networks, overcoming the basic limitations of traditional perceptrons that have challenged scientists for half a century.

## 1. Introduction

It is thought that we are at the beginning of a new industrial revolution, the so-called Industrial Revolution 4.0, in which Machine Learning will play a fundamental role in the development of new powerful intelligent systems to successfully process huge amounts of available information from different users to improve services and associated businesses [1]. Developing and optimizing machines capable of improving their operation through learning from information contained in data captured from the environment by electronic sensors are key research issues for the upcoming decades. The very concept of Machine Learning is taken from biological beings that adapt to the changing information provided by their senses. A new generation of machines is addressing the most important unsolved problem with artificial neural networks, that is, how to perform unsupervised learning as effectively as the brain [2]. This leads us to draw inspiration from biological researchers and transfer it to the artificial world of machines with the aim to produce a new generation of artificial neural networks (ANNs) that are necessary for the real development of the new industrial revolution.

Presently, the neuroengineering discipline focuses on modeling and applying artificial neural networks (ANNs) that mimic biological neurons and their interconnections, emulating their learning ability. Models are based on neurology research, a discipline that began about one hundred years ago with the studies of Ramón y Cajal [3], who pointed out that learning could produce changes in the communication between neurons and that these changes could be the essential mechanisms of memory. This property of neurons is now referred to as brain plasticity or Neuroplasticity, or the ability of the brain to modify its connections to produce changes in its communication.

Since then, biological research has accumulated an enormous amount of detailed knowledge about the structure and function of the brain. The elementary processing units in the central nervous system are called neurons and are connected to each other in an intricate pattern. Thus far, ANNs have not even come close to modeling the complexity of the brain, but they have been shown to be efficient at problems that are easy for a human but difficult for a traditional computer, such as image recognition and predictions based on past knowledge.

The origin of ANNs can be dated back to 1943, when neurophysiologist Warren McCulloch and mathematician Walter Pitts wrote a paper on how neurons might work [4]. In order to describe how neurons in the brain might work, they modeled a simple neural network using electrical circuits that are called perceptrons. The perceptron is a mathematical model of a biological neuron. While in actual neurons, the dendrite receives electrical signals (inputs) from the axons (outputs) of other neurons, in the perceptron, these electrical signals are represented by numerical values. At the synapses that connect the dendrites and axons, electrical signals are weighted in various amounts. This weighting is also modeled in the perceptron by multiplying each input value by a value called the weight *w*. An actual neuron activates and fires an output signal only when the total strength of the weighted input signals exceeds a certain activation threshold. This phenomenon is modeled in a perceptron by calculating the weighted sum of the inputs to represent the total strength of the input signals, and the step function *f(.)* is applied to the sum as an activation function to determine its output. As in biological neural networks, this output is inputted into other perceptrons.

After the McCulloch and Pitts perceptron, the first model of synaptic plasticity evolution was postulated by D.O. Hebb in 1949 and is commonly known as the Hebb rule [5]: “*When an axon of cell A is near enough to excite a cell B and repeatedly or persistently takes part in firing it, some growth process or metabolic change takes place in one or both cells such that A’s efficiency, as one of the cells firing B, is increased*”.

**Figure 1:**
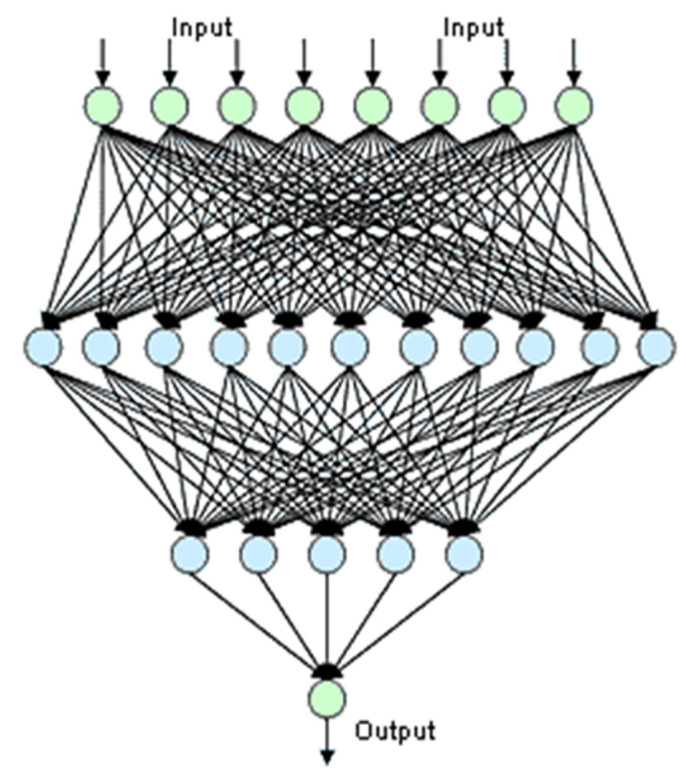
Typical connections of a Multilayer Perceptron with two hidden layers.

Research on perceptrons, with their learning and applications, was popularized in the 1960s and later in the 1980s, with the connection of perceptrons disposed into layers that are called first- and second-generation ANNs, respectively. In second-generation ANNs, so-called Multilayer Perceptrons (MLPs) or feedforward networks can be considered as the simplest but the most powerful artificial model of the biological “brain”, based on the simple model of neuron interconnection in layers (see Figure 1).

In its structure, we distinguish an output layer of output neurons that will usually be used to recognize classes; if an output is activated, the ANN recognizes the corresponding class as designed. The layer that feeds the output layer is called a hidden layer, which in turn is fed by another hidden neuron layer and so on till the first layer or input layer, which is fed by the input vector. An MLP is then designed by searching a matrix of weights *W* through an iterative process called training, the objective of which is minimizing the classification error of the universe of input vectors in such a way that when an input data vector feeds the ANN, it activates the output corresponding to the class of the data or corresponds to a desired output. In this last case, the network is applied for functional approximation; that is, the network is trained to perform an arbitrary nonlinear function *Y = F (X)* of the input vectors.

As illustrated in Figure 1, an MLP is physically composed of layers of aligned simple perceptrons which are fully connected in a feedforward way from layer to layer. The most relevant limitation, linear separability, is associated with each layer in this structure. This means that if we consider the input vector *X* as a point on a hyperspace, then each perceptron layer only can perform successfully if points are in convex regions limited by hyperplanes. Note that some logic functions such as the Boolean operators AND, OR and NOT are linearly separable problems, i.e., they can be performed using a single perceptron layer. However, not all logic operators are linearly separable. For instance, the XOR operator is not linearly separable and cannot be achieved by a single perceptron layer, since it is impossible to draw a line to divide the regions containing either 1 or 0. Although, even with this fundamental limitation, the perceptron initially seemed promising, it was quickly proven that a perceptron layer could not be trained to recognize many classes of patterns. This caused the field of ANN research to stagnate for many years, challenging scientists. This limitation was finally palliated with the use of more than one perceptron layer arranged in feedforward net with one hidden layer, considerably raising the system complexity, to form the MLP.

The first algorithm to train an MLP was called Backpropagation by Rumelhart, Hinton and Williams [6] and dates to 1986, although the technique was independently rediscovered several times, and had many predecessors dating back to the 1960s. It is a stochastic gradient descent method whose objective function is minimizing a function error like the Mean Square Error (MSE) between the MLP output during learning and the desired output.

Three years later, a Russian mathematician called George Cybenko [7] demonstrated that an MLP consisting of only one hidden layer and composed of an arbitrary number of artificial neurons was able to perform any nonlinear classification or continuous function approximation if the proper weight matrix *W* was found. Thus, MLPs have since then been referred to as Universal Approximators.

In 1990, Ruck et al. [8] demonstrated that, when modeled by a sigmoidal activation function in [0, 1], the MLP output provides an inherent network estimation of the a posteriori probabilities of the inputs. Such a Multilayer Perceptron can be considered as an approximator to the Bayes Optimal Discriminant Function. 

The ANNs referred to so far are called rate-coding artificial networks, as they model only the amount of excitation or the rate of short electrical impulses that biological networks receive and produce. These pulses, called action potentials or spikes, have an amplitude of about 100 mV and typically a duration of 1–2 ms. The form of the pulse does not change as the action potential propagates along the axon. A chain of action potentials emitted by a single neuron is called a spike train—a sequence of stereotypical events which occur at regular or irregular intervals. Since all spikes of a given neuron look alike, the form of the action potential does not carry any information. Rather, it is the number and the timing of spikes which matter. The action potential is the elementary unit of signal transmission. Action potentials in a spike train are usually well separated. Even with a very strong input, it is impossible to excite a second spike during or immediately after the first one. The minimal distance between two spikes defines the absolute refractory period of the neuron. The absolute refractory period is followed by a phase of relative refractoriness where it is difficult, but not impossible, to excite an action potential. The effect of a spike on the postsynaptic neuron can be recorded with an intracellular electrode, which measures the potential difference between the interior of the cell and its surroundings. This potential difference is called the membrane potential. Without any spike input, the neuron is at rest, corresponding to a constant membrane potential. After the arrival of a spike, the potential changes and finally decays back to the resting potential. If the change is positive, the synapse is said to be excitatory. If the change is negative, the synapse is inhibitory.

At rest, the cell membrane already has a strong negative polarization of about −65 mV. An input at an excitatory synapse reduces the negative polarization of the membrane and is therefore called depolarizing. An input that increases the negative polarization of the membrane even further is called hyperpolarizing.

The spikes train resembles a digital transmission, so the exact timing of spikes should play a role. It is important to remember that there is a physical limit on how fast a neuron can fire. The ANNs that model this behavior, that is, those that consider time in their design, are called Spiking Neural Networks (SNNs) and have at least a similar applicability to static ones, although they are usually more difficult to train, showing generally more efficiency in the applications where time plays an important role. Research on SNNs and their learning was popularized in the 1990s, in what is called the third generation of ANNs.

ANN structures can be cascaded to form more complex hybrid networks or Deep Networks. In general, neural networks that are capable of tackling more abstract problems by this interconnection or another method are called Deep Learning Networks. They integrate artificial neural systems that are trained together or separately but work together to perform a more sophisticated global task than a single hidden layer or individual networks. Nevertheless, they are also claimed to be bioinspired, as they are structurally more complex, like biological neural networks. Some authors claim that they correspond to the fourth generation of ANNs, as they introduce the novelty of achieving Deeper Learning by physically Deeper Networks, while others just consider them as part of the second generation. Bioinspired neural network models help to understand autonomous adaptive intelligence [9], and the author of this paper supports that Deeper learning, that is, a higher level of abstraction, will be achieved not only based on more complex networks, but also based on modeling advances from neurologists in the neuron models. This is called Bioinspired Deep Learning, that is, simulating higher-order characteristics of the biological neurons in the node and weight updates of Machine Learning and so achieving a global higher order level of abstraction in performing information extraction on Big Data training sets.

Up to now, introductory concepts and definitions briefly summarizing the fundamental state of the art of biological models already successfully implemented have been presented. The main aim of this work is to support the relevance of bioinspiration for the future of these models and therefore of Machine Learning and so too the incipient associated new industrial revolution by modeling advanced neuronal properties in rate-coding ANNs. The selected properties are intrinsic plasticity, metaplasticity and lateral inhibition that allow for competitive learning and performance. The powerful characteristics that they can provide to current ANNs pave the way to a potential new generation of ANNs characterized by overcoming the fundamental limitations of current basic components like classical perceptrons and linear separability. These ANNS are scalable and able to perform continuous learning for deeper, more efficient, and more powerful Machine Learning.

The arrangement for the rest of this article is as follows: In Section 2, the author describes the bioinspired parameters and properties applied to achieve the results in a way that can be easily implemented. In Section 3, the most relevant implementation results of the bioinspired improvements so far are presented, ending with a novel proposal: the Competitive Perceptron. In Section 4, a proper discussion is developed, and the conclusions are finally summarized in Section 5. 

## 2. Materials and Methods

In this section, the author describes the bioinspired parameters and properties applied to achieve the results in a way that can be easily implemented. Appropriate references are provided.

### 2.1. Plasticity and Learning

Neurons are said to be plastic because they can change the value of their synaptic strengths. In an artificial model, the weight matrix *W* represents this plasticity, and the memory of the ANN and its variation, Δ*W*, therefore represent learning, i.e., its memory variation in order to adapt and improve performance. It is therefore the fundamental parameter common to all networks.

### 2.2. Long-Term Potentiation and Depression

Bienestock, Cooper, and Munro [10] postulated that a biological neuron possesses synaptic modification thresholds (LTP/LTD threshold), which dictate whether the neuron’s activity at any given instant will lead to strengthening or weakening of its input synapses. Upregulation—incrementing reinforcement of synaptic efficacy—is termed Long-Term Potentiation (LTP), whereas downregulation—decrementing inhibiting—is known as Long-Term Depression (LTD). LTP and LTD are believed to be fundamental to the storage of memory in the brain and hence to learning [11].

### 2.3. Metaplasticity

**Figure 2:**
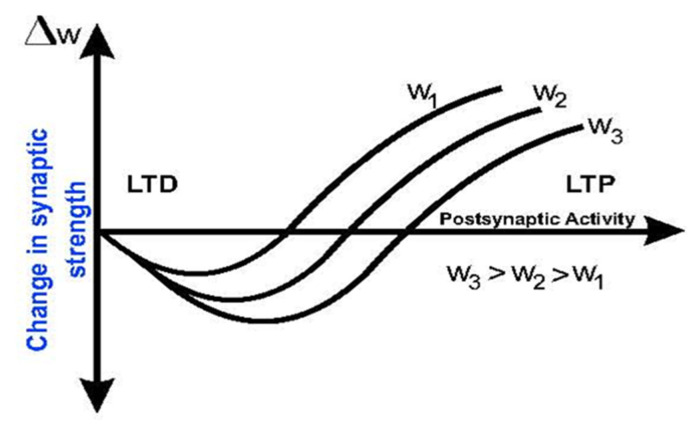
Family of curves that illustrate metaplasticity.

In 1996, Wickliffe C. Abraham [12] introduced the concept of biological metaplasticity that is now widely applied in the fields of biology, neuroscience, physiology, neurology and others like bioinspired Artificial Intelligence. The prefix “meta” comes from Greek and means “beyond” or “above”. It indicates that metaplasticity is a higher-order form of synaptic plasticity: “the plasticity of synaptic plasticity”. Figure 2 shows a family of curves that illustrate metaplasticity. Each curve indicates the plasticity respective of the neuron’s activation.

Cell firing or postsynaptic activity has been reported to variably upregulate or downregulate the subsequently induced LTP and LTD. Plasticity depends on postsynaptic activation, and in Figure 2, it can be observed that for a given postsynaptic activity, a lower value of weights corresponding to a lower previous activity presents a higher Δ*w* (higher learning), so they are able to “learn” more [13]. 

Metaplasticity indicates a higher level of plasticity, expressed as a change or transformation in the way that synaptic efficacy is modified. Metaplasticity is then defined as the induction of synaptic changes that depend on prior synaptic activity in [12], where Abraham speculates that “…*prior synaptic activation can leave an enduring trace that affects the subsequent induction of synaptic plasticity*”, and in [14], where it may play an important role in maintaining synaptic strengths within a dynamic range that is optimal for the stability of the learning process (to prevent saturation) (homeostasis). Metaplasticity can potently influence information storage properties (memory) at the synaptic level. Note that in biology, memory seems to happen at different levels, whereas in ANN models, usually there is no other kind of memory.

D. Andina et al. [13] modeled it through a variable learning rate in the general equation of MLP learning, drastically improving the learning time and performance of an MLP.

(1) $$\Delta w_{ij}^{l}=w_{ij}^{l}\;(t+1)-w_{ij}^{l}\;(t)=-\eta (I_{ij})\;\frac{\partial E(W)}{\partial w_{ij}^{l}}$$

where *w* is the synaptic weight;

*l* = layer counter;

*I* = input counter;

*J* = neuron counter;

*t* = training iteration counter (one input pattern by iteration);

*η* = learning rate ⋿ [0, 1], a tuning parameter of the amount of learning by iteration;

*W* = set of all synaptic strengths in the MLP;

*E(W)* = error function.

Thus, each artificial synaptic weight variation that each pattern produces during learning, Δ*w,* is higher for a lower activity—a lower postsynaptic activity—and vice versa. Artificial metaplasticity (AMP) is then defined as:

*“A learning procedure that induces greater modifications in the artificial synaptic weights W with less frequent patterns as they produce less prior firing than frequent patterns”* [13].

This model then makes the artificial neurons learn more from the low probability input training patterns than from frequent ones. This can be modeled with a variable learning rate *η (I_ij_)* depending on the distribution of training input patterns. The universality of improvements to implementing artificial metaplasticity has been demonstrated in very different multidisciplinary applications, such as breast cancer diagnosis, wood classification, credit scoring, etc. [15,16,17,18]. 

### 2.4. Intrinsic Plasticity

**Figure 3:**
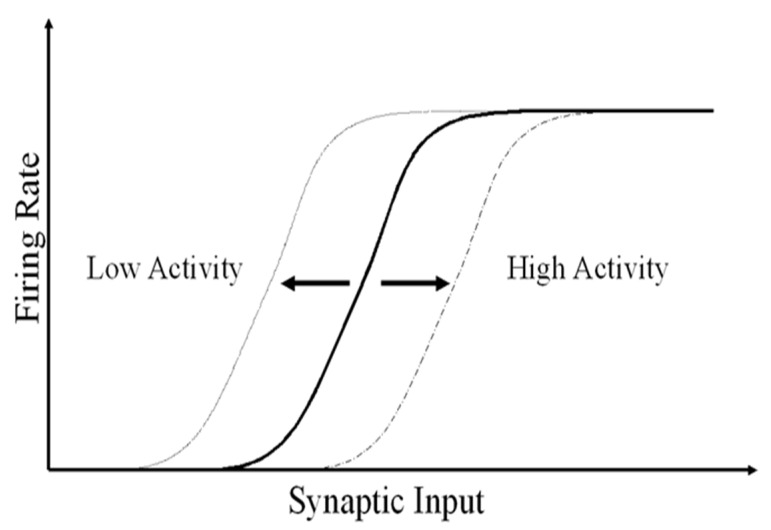
Intrinsic plasticity of the neuron’s activation function according to previous synaptic levels of activity. Higher activity shifts the sigmoid rightwards and lower activity shifts the sigmoid leftwards.

In the cellular soma, intrinsic plasticity is a homeostatic mechanism that regulates the position (rightward shift) of the neuron’s activation function according to previous levels of activity, preventing nullification or saturation of *w*. Higher activity shifts the sigmoid rightwards and lower activity shifts the sigmoid leftwards (see Figure 3). It is called homeostatic plasticity as it serves to stabilize the network activity, preventing nullification or saturation of neuron responses. 

In a novel model, Peláez and Andina [19] implemented intrinsic plasticity in a KLN, a neural network model inspired by thalamo–cortical interactions and the Koniocortex of biological brains. To model learning, they applied a plausible hypothesis of synaptic weight modification that empirically yields plasticity curves that present the already described metaplasticity and also intrinsic plasticity. This model is the bioinspired probabilistic equivalent to the incremental version of the Grossberg’s pre-synaptic rule [20,21,22], where Δ*w* is calculated in each iteration as:

(2) $$\;\Delta w_{ij}=\eta I(O-w_{ij})$$

where *O* and *I* represent the postsynaptic and presynaptic action potential probabilities, respectively, and η is the learning factor. Then, the synaptic weight between two neurons approximates the conditional probability of the output neuron’s firing, given that the neuron has previously fired:

(3) $$\;\Delta w_{ij}=\eta I(O-w_{ij})$$

The neuronal activation function of each neuron *j* is modeled as a sigmoid:

(4) $$O_{j}=\frac{1}{1+e^{-k({net}_{j}+0.5-2s_{j})}}$$

where *net_j_* is the net input of neuron *j* calculated as the sum of inputs weighted by synaptic strengths *W^j^*,

(5) $${net}_{j}=\sum_{i}^{N}w_{ij}x_{i}=\left|\left|\underline{W^{j}}\underline{I}\right|\right|$$

and *s_j_* is the shift of the activation function and *k* is a sigmoidal-compressing factor. The range of *s_j_* is 0 < *s_j_* < 1, and *k* ϵ R controls the sigmoid slope and its value depends on the type of neuron. The intrinsic plasticity or shift *s_j_* of the sigmoid is determined in each iteration *t* by the following equation:

(6) $$s_{j}(t)=\frac{\nu O_{t}+s_{j}(t-1)}{1+\nu }$$

Here, *ν* ≥ 0 a velocity parameter that tunes the amount of shift in each iteration.

Via the effect of the shift *s*, the firing probability of neurons adapts to activation, making very active neurons moderate their activity and inactive neurons become more active as the activation function gradually shifts rightwards or leftwards and thus balancing the activation. In the case of highly activated neurons, *s* increases, making the activation function shift rightwards so that the output of the neuron will be downregulated in the future. In the case of less active neurons, the activation function shifts leftwards, and the neuron increases its firing. Note that when both the shift and the output at iteration *t* − 1 are equal in (6), the shift at time *t* continues to have the same value of the shift at time *t* − 1.

Biological mechanisms of plasticity and metaplasticity are still unclear [23]; new models may shed new light, corresponding to artificial advancements in modeling.

### 2.5. Competitive Learning by Lateral Inhibition

Competitive learning is a form of unsupervised learning in artificial neural networks in which nodes compete for the right to respond to a subset of the input data. A variant of Hebbian learning, competitive learning works by increasing the specialization of each node in the network. It is well suited to finding clusters within data. Competition occurs naturally due to inhibition between neurons; when one activates, it inhibits others. Although a winning neuron is identified by calculation in many artificial neural network models, in biological neural networks such as KLNs, the winning neuron emerges in a dynamic process with lateral inhibition, a phenomenon in which a neuron’s response to a stimulus is inhibited by the excitation of a neighboring neuron as an important driving force. In this study, we consider that the homeostatic properties such as metaplasticity and intrinsic plasticity contribute to Winner-Takes-All (WTA) dynamics and make competition possible when driven by lateral inhibition. In combination with intrinsic plasticity, Peláez et al. [19] showed that biological neurons can perform the WTA algorithm, as presented in the results.

## 3. Results

This section presents the most relevant implementation results of the bioinspired improvements presented so far and ends with a novel proposal. In paper [19], Peláez and Andina presented the Koniocortex-Like Neural Network (KLN), a fully biologically plausible ANN inspired by biological koniocortex cells, a category of neural networks that resembles the cerebral koniocortex. In it, synaptic plasticity, metaplasticity, intrinsic plasticity and lateral inhibition are orchestrated to produce relevant results as associative NNs, classification and general unsupervised NNs. The KLN incorporates all proposed bioinspired advancements that have been illustrated in this paper and is also those applied to calculate the results. Then, a novel simplified Competitive Perceptron model is proposed as a case whose results illustrate the advances compared to MLPs. It aims to capture the advantages of the KLN and transfer them the classical MLP by replacing the perceptron used so far in its construction by a new competitive perceptron structure that learns through the bioinspired learning algorithm of the presynaptic rule. The drastic and fundamental improvements that it introduces are then discussed to support the conclusions. 

### 3.1. Brief Description of the KLN

The already defined Koniocortex-Like NN [19] was proposed to be applied as a general-purpose Machine Learning system for successful multidisciplinary real-data applications. KLNs are designed to follow the biological reality as closely as possible. 

The koniocortex, also known as the granular cortex, is located in the cerebral cortex. Both names (koniocortex and granular) refer to a cortex with a grainy texture (*konia* is a Greek word meaning “dust”). Brodmann areas 1–3 of the somatic sensory cortex, area 17 of the visual cortex, and area 41 of the auditory cortex belong to the koniocortex [19]. All these areas behave like topographic maps that change their boundaries and receptive fields according to the sensory experience [24,25].

**Figure 4:**
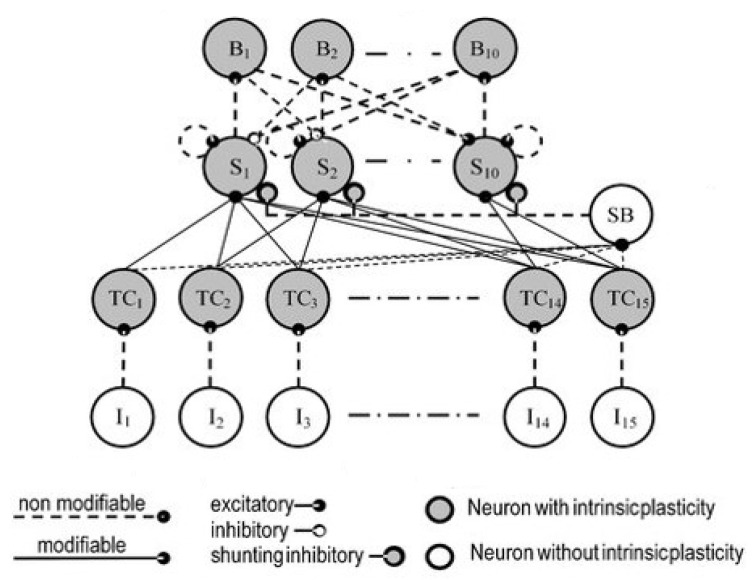
The KLN proposed in [19].

Figure 4 presents the KLN initially modeled in [19]. I1 to I15 receive input vectors and their outputs feed corresponding neurons TC1 to TC15, named after thalamocortical neurons. TC neurons act as pre-processing units, performing normalization of input components together with SB, a shunting (or divisive) basket neuron that performs the arithmetical summation of its inputs (TC outputs), dividing the activation of its target neurons (the S neurons) by this quantity. TC neurons, through intrinsic plasticity, extract the mean component of the input set. S1–S10 model spiny stellate neurons that perform intelligent processing, are the main parts engaged in competition and are also endowed with intrinsic plasticity. Each S neuron has a connection to itself (autapse). This kind of recurrent connection is rare in general neurons but necessary in the KLN model to efficiently mimic the biological reality and indeed is present in the koniocortex. B1–B10 model basket neurons are endowed with intrinsic plasticity and non-modifiable synapses (as usual in real inhibitory neurons) and perform inhibition and thus competition, the most relevant mechanism for classification. Competition occurs naturally due to inhibition between neurons, and it is detailed below.

### 3.2. Lateral Inhibition

**Figure 5:**
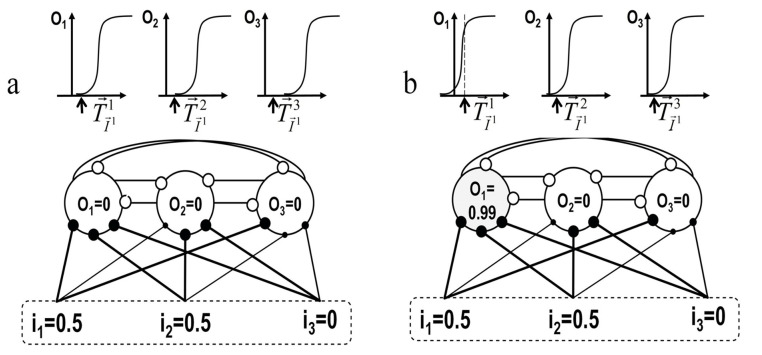
Implementation of lateral inhibition. (**a**) Even when no neuron has yet reached the firing threshold, they experiment with intrinsic plasticity so that (**b**) sigmoids corresponding to activation functions shift leftwards until one of the neurons fires, preventing the remaining neurons from firing due to lateral inhibition.

Competition in the KLN is forced by lateral inhibition, so that the WTA neuron is set to one and the others to zero. In the KLN, competition occurs naturally owing to the interplay between inhibitory interneurons and synaptic and intrinsic plasticity. The dynamics of lateral inhibition are schematically represented in Figure 5. 

This lateral inhibition network has the following characteristics:

(a) Each neuron laterally inhibits its neighbors.
(b) Each activation function has steep slopes.
(c) Intrinsic plasticity regulates neurons’ activations.
(d) The presynaptic rule is used for learning synaptic weights. The most activated neuron emerges from the internal dynamics of the network in which each neuron acts without any kind of external supervision.

The process is as follows: Initially (Figure 5a), weights are random and small. Due to this, the net input is also small and incapable of producing a significant output. A null output in all neurons makes all activation sigmoids shift leftwards (according to Figure 3 and Equations (6) and (4)) so that the most active neuron eventually fires (O1 in the example of Figure 5b), preventing remaining neurons from firing due to lateral inhibition. However, the winning neuron will not permanently be the winner, because once a certain neuron wins, its sigmoid tends to shift rightwards according to intrinsic plasticity. Note that inhibitory connections do not undergo weight variations (in this case, inhibitory connection weights were set to 1). An improvement in the operation of this lateral inhibition network (especially when leading with randomly presented patterns) was obtained in the KLN by using autapses (recurrent positive connections).

The first results of KLNs [19,25] showed that they can be successfully applied as associative NNs and learning vector quantization NNs with two novel and extremely relevant properties: continuous unsupervised learning and scalability. These are two challenging concepts in present NNs [2]. In [25], a KLN is applied for the first time as an unsupervised learning classifier, achieving better results than all other state-of-the-art classifiers, including supervised learning designs. The application was a complex one: the classification of the WBCD Breast Cancer Database. Similar results were also achieved for completely different applications: business data in [26,27] and for cardiac arrhythmia classification in [28].

### 3.3. The Competitive Perceptron

The KLN was developed following biological plausibility in the whole model. However, in an artificial engineering model, it can be simplified while maintaining the characteristics that have helped them succeed. The result is not biological and does not correspond to the koniocortex, but it has the most relevant properties of a KLN. In summary, what is going to be engineered is:

*“…a single layer perceptron with normalized inputs, lateral inhibition in the processing neurons and trained by the presynaptic rule.”* [19].

**Figure 6:**
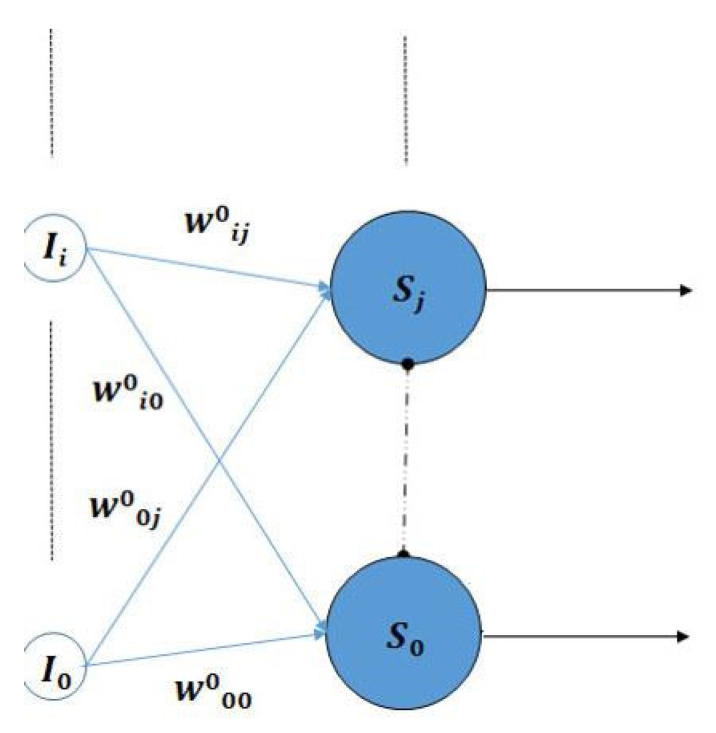
The *Competitive Perceptron* is a single-layer perceptron that includes intrinsic plasticity in the neuron activation function and lateral inhibition connections among the neurons in the same layer (represented by a dashed line ended in a black dot) to allow for WTA competition.

By directly implementing this last sentence, we obtain a single layer perceptron with the structure in Figure 6, where the dashed line ending in a black dot represents lateral inhibition among all neurons of the layer. These neurons also implement intrinsic plasticity in their activation functions (Equation (4)) and present metaplasticity by being trained with the already described presynaptic rule in Equation (2). Let us call it Competitive Perceptron (CP). It has been designed to have the same learning and performance properties as the KLN, that at the moment, it has been able to perform many different complex classifications and its versatility allows it to be used in a supervised and unsupervised way, as an associative and learning vector quantization network among others [25,26,27].

**Figure 7:**
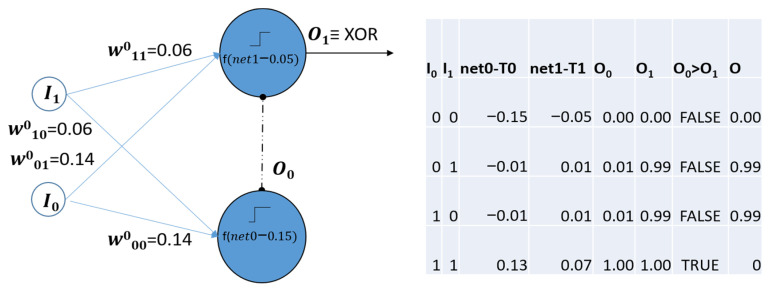
XOR function performed by the simplest two neurons in the Competitive Perceptron.

To show its potential, let us illustrate as a result a fundamental advance at the neuronal level over the classical perceptron. The CP in Figure 7 was successfully trained to perform the XOR function, as can be confirmed through the parameter values in the right-hand side of the figure. Thus, it does not present the linear separability limitation of classical perceptrons.

Here, *net_j_* is given by (5), *T_j_* = 2*s_j_* – 0.5 (see Equation (4)), representing the activation thresholds of artificial neurons *j*, and *O_j_* is their respective outputs given by (4) with *k* = 500. After successful training, for the obtained *W* results, *T*_0_ *=* 0.15 and *T*_1_ *=* 0.05, as deduced from the table in Figure 7.

Note that to determine the *WTA* winner neuron, the plausible comparison is total neurons excitation *net_j_* − *T_j_*, instead of the corresponding *O_j_*. The sigmoidal activation function is very steep (*k =* 500), as occurs in biological neurons and the KLN. Thus, although *O*_0_ and *O*_1_ may seem equal within a few decimal precision (see the last file of the table in Figure 7), working with sufficient precision, *O*_0_ *= sigmoid (*0.13*) > sigmoid (*0.07*) = O*_1_. The CP inherits this property from the KLN as the rest of fundamental ones. 

In MLPs, adding more layers or even more neurons in hidden layers does not necessarily lead to learning and performance improvements. A high number of neurons in a layer or more than one hidden layer can even degrade the performance. In the CP, as in the KLN, adding more neurons improves learning and performance [19]. As the MLP is probably the most applied ANN up to now and is nowadays frequently used also as the final part of Deep Networks that produce the main state-of-the-art improvements, the fact that the CP improves the scalability and function approximation at the neuron level is an advancement of major relevance.

### 3.4. Limitations of This Study

It is fundamental to note that, although bioinspired, rate-coding ANNs, such as the Competitive Perceptron presented, do not take in account the time variable in trains of pulses in communication among artificial neurons. Only the effect of the amount of excitation or rate of short electrical impulses that biological networks receive and produce may then be reproduced by them.

### 3.5. Future Research

It is proven that MLPs are universal approximators. The CP seems to have the same property, with a much simpler structure of one single layer, and no counterexample has been found at the moment. Thus, a mathematical proof such as in [7] is expected to be presented in the near future. Its hypothetical universality, scalability, and continuous learning make it worthy of research in comparison to MLPs and many other classes of rate-coding ANNs. Note that the CP is just a result of implementing the presented bioinspired properties in MLPs. 

## 4. Discussion

This paper proposes the artificial implementation of three relevant properties of biological neurons to upgrade the characteristics of present rate-coding ANN models: metaplasticity, intrinsic plasticity, and lateral inhibition. Metaplasticity has been shown to drastically improve the training phase of ANNs [13,14,15,16,17,18]. It can be implemented not only in MLPs by Equation (1) but also by applying presynaptic rule learning as stated for the KLN and the CP, or even both, as the concept of learning more from infrequent patterns than frequent ones can be applied to any class of rate-coding ANNs as Self Organization Maps (SOMs), Radial Basis Function Networks, or Convolutional Neural Networks [29,30,31]. Many other contributions for the further development of brain-like intelligence models can be found by many other approximations that are currently being researched [32,33]. A thorough comparison would be a matter for an extensive review paper [34].

Intrinsic plasticity is probably responsible for the possibility of continuous learning shown by the KLN, as it avoids the saturation of neuron activation functions and thus the corresponding stagnation of learning. Implementing the concept of a shift in the activation function according to successful or unsuccessful classification is straightforward to generally apply in ANNs, as long as artificial neurons are provided for the activation function. 

The competitive dynamics that lateral inhibition introduces by implementing WTA competition among neurons mimic natural classification, simplifying the competition and thus contributing to the scalability of the resulting design. The combination of them also incorporates new emerging capabilities, as illustrated by the case of the CP, where the combination of lateral inhibition and intrinsic plasticity allows the Competitive Perceptron to avoid the limitation of linear separability. All these improvements, if transferred to present ANNs, can pave the way to a new generation of neural networks able to come closer to fulfilling the objective of performing unsupervised learning as effectively as the brain, recognized as the current challenge in ANN research by the scientists most involved in the present industrial revolution like Hinton [2], or at least contribute to it. This is yet to be tested and proven by the researchers that implement them. 

To support this hypothesis, the Competitive Perceptron, engineered from KLN implementation, is presented; that is, the CP does not model biological reality but it is inspired by its mechanisms. It is a single-layer perceptron that includes the proposed advances with the potential of non-linear separability, continuous learning, and scalability. Thus, it is suitable to contribute to building efficient Deep Networks, overcoming the basic limitations of traditional perceptrons, such as the inefficient scalability and linear separability problems. Its potential and simplicity in comparison with MLPs, which are probably the most applied ANNs and are presently used as the last stage of many Deep Learning Networks, can allow it to improve countless applications where MLPs are being applied successfully. Although this is not proven to be a definitive solution, it is evident that implementing these bioinspired characteristics can improve present designs at the neuronal level. The limits of its implementation in learning and performance are still to be reported by those scientists that study it.

## 5. Conclusions

The incipient industrial revolution requires advances in Machine Learning to allow models to mimic the efficiency of biological learning. For this purpose, new engineering models of artificial neuronal structures and learning are a relevant line of research. This paper shows that bioinspiration from new, relevant biological findings can pave the way to this objective. To illustrate this, the author has reviewed the characteristics and results of Konicortex-Like Neural Networks, models that can be trained in an unsupervised and supervised mode, such as associative memory and vector quantization, and show relevant properties such as continuous learning, scalability, and presumed universality of classification. When engineered to solve real multidisciplinary problems using real data, it has produced machines that compete with or improve the state-of-the-art results. A new kind of ANN, called the Competitive Perceptron NN, is finally presented to illustrate how the proposed bioinspired characteristics may drastically improve MLPs and therefore all MLP applications, from the simplest models to the Deep Networks that are presently leading the improvements in Machine Learning and are where the bioinspired improvements presented in this paper can also be implemented at the artificial neuron level.

## Data Availability

No new data were created or analyzed in this study. Data sharing is not applicable to this article.
